# Single-cell sequencing analysis fibrosis provides insights into the pathobiological cell types and cytokines of radiation-induced pulmonary fibrosis

**DOI:** 10.1186/s12890-023-02424-5

**Published:** 2023-04-28

**Authors:** Zhiyong Sun, Yutao Lou, Xiaoping Hu, Feifeng Song, Xiaowei Zheng, Ying Hu, Haiying Ding, Yiwen Zhang, Ping Huang

**Affiliations:** 1 Clinical Pharmacy Center, Department of Pharmacy, Zhejiang Provincial People’s Hospital, Affiliated People’s Hospital, Hangzhou Medical College, Hangzhou, Zhejiang China; 2 College of pharmacy, Zhejiang University of Technology, Hangzhou, Zhejiang China; 3 Zhejiang Cancer Hospital, Institute of Basic Medicine and Cancer (IBMC), Chinese Academy of Sciences, Hangzhou, 310022 Zhejiang China

**Keywords:** Radiation-induced pulmonary fibrosis, Single-cell RNA-seq, T cells, B cells

## Abstract

**Background:**

Radiotherapy is an essential treatment for chest cancer. Radiation-induced pulmonary fibrosis (RIPF) is an almost irreversible interstitial lung disease; however, its pathogenesis remains unclear.

**Methods:**

We analyzed specific changes in cell populations and potential markers by using single-cell sequencing datasets from the Sequence Read Archive database, PERFORMED from control (0 Gy) and thoracic irradiated (20 Gy) mouse lungs at day 150 post-radiation. We performed IHC and ELISA on lung tissue and cells to validate the potential marker cytokines identified by the analysis on rat thoracic irradiated molds (30 Gy).

**Results:**

Single-cell sequencing analysis showed changes in abundance across cell types and at the single-cell level, with B and T cells showing the most significant changes in abundance. And four cytokines, CCL5, ICAM1, PF4, and TNF, were significantly upregulated in lung tissues of RIPF rats and cell supernatants after ionizing radiation.

**Conclusion:**

Cytokines CCL5, ICAM1, PF4, and TNF may play essential roles in radiation pulmonary fibrosis. They are potential targets for the treatment of radiation pulmonary fibrosis.

## Introduction

Lung cancer is one of the most common malignant cancers. It has extremely high incidence and very low survival rates and accounts for almost one-quarter of all cancer-related deaths[1]. Radiotherapy (RT) is an effective treatment for both primary and secondary lung cancer [2–4]. Novel techniques such as stereotactic ablative body radiotherapy have been introduced; however, ionizing radiation can also induce serious side effects that limit the efficacy of RT [5, 6]. Radiation-induced lung injury is a common toxicity effect of RT [7, 8] and includes acute radiation responses, radiation pneumonitis in the early stage, and pulmonary fibrosis (PF) in the late stage [8]. Fibrosis involves excessive deposition of connective tissue components outside the cell owing to inflammation or injury, ultimately destroying the physiological structure and leading to organ dysfunction [9, 10]; To date, its pathogenesis remains unclear.

The analysis of gene expression in lung tissue cells is a suitable approach to decipher pathological changes in RIPF; however, there is a high degree of cellular heterogeneity in these cells. The advent of single-cell RNA-sequencing (scRNA-seq) technology can disclose new cell subpopulations and enable further exploration of gene regulation mechanisms, revealing the genetic and functional heterogeneity of each cell. We explored the mechanisms of RIPF by analyzing different cells in lung tissue samples using the RIPF scRNA-seq technology transcriptome dataset.

B and T cells reportedly play an integral role in the pathogenesis of PF[11]. Lymphocytes are located in the areas of fibrosis adjacent to fibroblastic lesions, suggesting their involvement in PF [12, 13]. B-cell aggregates are generated following lung infection and injury, where microbial antigens activate B cells which then release pro-fibrotic and pro-inflammatory factors (MMP-7, IL-8, IL-6, and VEGFA) to catalyze the migration and activation of fibroblasts, thereby promoting intercellular communication between B cells and fibroblasts [14]. B-cell activating factor is enriched in the lungs and blood of patients with IPF. CD20-targeted therapy (removal of B cells) improves lung function [15, 16]. B cells are reportedly involved in the pathogenesis of PF[17, 18].

Fibroblasts are crucial cells in PF[19]. Pulmonary fibroblasts secrete pro-apoptotic molecules that produce an apoptosis-inducing microenvironment, consequently inhibiting the ability of T cells to migrate, such that fibroblastic foci are virtually devoid of T lymphocytes[20]. This prevents the immune system from eliminating fibroblasts or myofibroblasts[21]. All these factors have been investigated in idiopathic PF; however, there remains a lack of studies on RIPF.

In this context, we explored the potential mechanisms involved in T and B cells by profiling RIPF single-cell transcriptome sequencing findings.

## Materials and methods

### Single-cell transcriptome sequencing source and pre-processing

The study used the RIPF-associated single-cell transcriptome sequencing dataset in the Sequence Read Archive (SRA, https://www.ncbi.nlm.nih.gov/sra) database, PRJNA768942. Single-cell RNA-Seq was performed on single-cell suspensions generated from two control and two 150 days post-irradiated (20 Gy) irradiated mouse lungs with fibrosis. Used a Mark I Gamma Cell Irradiator (JL Shepherd & Associates, San Francisco, CA) with filter removed. This action resulted in a dose rate of 343 cGy per minute.

The SRA files of the PRJNA768942 dataset were converted to fastq files in SRA Toolkit software (version 2.11.0), and Cell Ranger software (version 6.0.2) was used to complete the cellranger count process to obtain single-cell mRNA expression data.

In the R environment (version 4.0.3), each sample of the single-cell transcriptome sequencing dataset underwent quality control using the Seurat package under the following conditions: nFeature_RNA 200–6,000%, mt < 15%, and nCount_RNA > 10. The QC’d single-cell sequencing samples were combined separately, and the NormalizeData and ScaleData function was used for logarithmic normalization and linear regression scaling of the data, and the principal component analysis (PCA) method was then used to characterize all gene expressions. Dimensionality reduction analysis was performed, and Harmony R package was combined to correct batch effects between different samples. FindNeighbors function was used to construct a graph of the dimensionality reduction results. Clustering was then performed using the FindClusters function.

### Analysis of gene expression differences between T cells and B cells

Seurat objects of T and B cells were separately extracted using the Seurat package in the R4.0.3 environment and combined using the Harmony package to recalibrate the batches and clusters.

The FindMarkers function was used to find the differential genes between T cells and B cells in the disease and control groups, respectively, and the genes with mean log2FC absolute values > 1 and BH corrected p-values < 0.05 were selected, and biological function enrichment analysis was performed using the clusterprofiler package. The ten functions with the smallest p-values were selected for visualization; PPI was then constructed in the STRING v11.5 database (https://cn.string-db.org/), and the network was visualized using the ggraph package and tidygraph package.

### Animal experiments

Sprague–Dawley rats (male, 7-week-old, 200 ± 20 g) were obtained from the Center for Experimental Animals, Zhejiang Chinese Medical University. The protocol was approved by the Experimental Animal Management and Ethics Committee of Zhejiang University of Traditional Chinese Medicine (Approval No.: IACUC-20210315-09). Animals were randomly divided into two groups: the control group and 30 Gy group. There were 8 rats in the control group and 8 rats in the irradiated group. 60 days after irradiation, all rats were sacrificed by intraperitoneal injection of an overdose of 1% pentobarbital sodium (150mg/kg). Death was confirmed by checking for cardiac arrest, after which the rat was observed for 10 min. After ensuring that there were no signs of recovery, tissues were collected from the rats. Rats were irradiated at the Irradiation Center of the Zhejiang Cancer Hospital.

The rats were anaesthetized using 1% pentobarbital sodium by intraperitoneal injection (40mg/kg), after anesthesia, the rats were placed supine, the limbs were fixed so that the chest was fully exposed, and a professional small animal radiation irradiator SARRP3(201,632,210,096, XStrahl, USA) was used to irradiate the whole chest with a single 30 Gy irradiation area of 3.5 cm*4 cm, i.e., the upper part of the rat to the two axillae and the lower part to the sternal process was aligned with the irradiation field, and the rest of the rat was shielded by 10 cm thick lead bricks with a source target distance of 100 cm and a dose of 230 cGy/min.

### Histological staining

Rats were euthanized in the 2nd month after irradiation, and fresh lung tissue was harvested and cut into paraffin sections. The sections were then subjected to, immunohistochemical (IHC) (YT4002: RuiYing Biotechnology Company, China, A3694, A5597: ABclonal Technology Co.,Ltd., and 60291-1-1lg: Proteintech Wuhan Sanying, China), hematoxylin-eosin (H&E) and Masson’s trichrome staining (Wuhan Pinuofei Biological Technology Co., Ltd., C h6ina) to assess the lung structure and protein expression.

### Determination of cytokine cytokines

According to the manufacturer’s instructions (EK1129, EK189, and EK182HS, ELK Wuhan Biotechnology CO., Ltd., China), enzyme-linked immunosorbent assay (ELISA) kits were used to examine serum levels of CCL5, ICAM1, PF4, and TNF-α in cell culture supernatants of BEAS-2B(CL0044, Hunan Fenghui Biotechnology Co., Ltd) at the 48th hour after 10 Gy irradiation. All samples were measured in duplicates.

## Results

### ScRNA-seq analysis

PCA and t-SNE analyses were performed. Depending on the expression of maturation markers, the cells were classified into 11 different cell types based on cell type-specific gene expression (Table 1). Cells in the two datasets were separately arranged using the following threshold conditions: average expression proportion of markers in each clustering subgroup > 30% and average expression value > zero. Annotations with overlapping subgroups were selected for annotation with a greater proportion of marker expression.

**Fig. 1:**
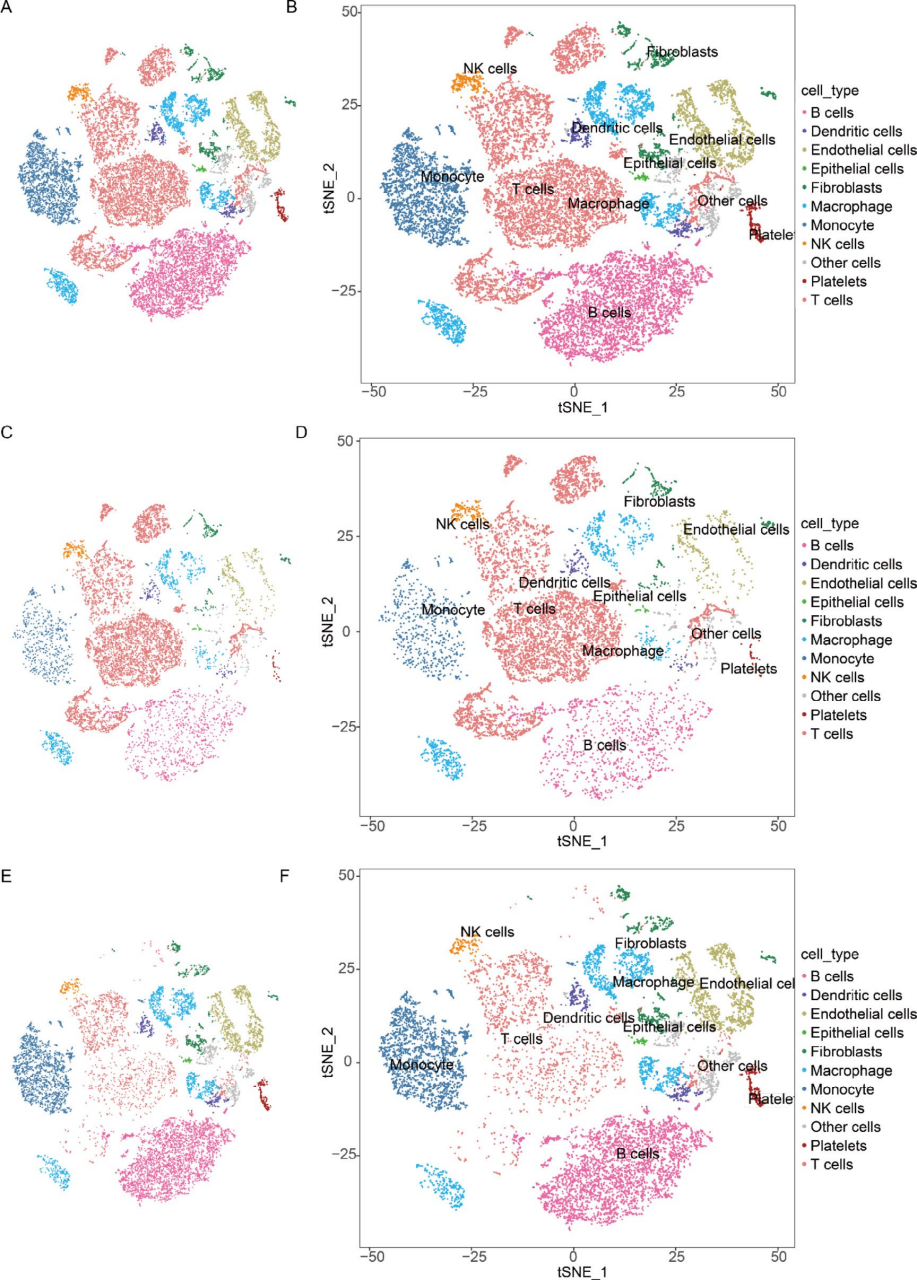
Single-cell RNA-seq analysis of radiation-induced pulmonary fibrosis in rats. SNE of the 19,879 cells analyzed in this study, with each cell color-coded for the associated cell type and the number of transcripts detected in that cell. Canonical correlation analysis was performed in R4.0.3 environment and then visualized via the above-mentioned nearest-neighbor graph, constructed in conjunction with the tSNE method. Cells were clustered using a graph-based shared nearest-neighbor clustering method, using canonical cell markers to label clusters by cell identity as indicated in the graph. Cell types were classified as indicated: (A, B) A t-SNE plot of 19,879 cells isolated from lung tissue, exhibiting 11 identified clusters; (C, D) t-SNE of normal mouse lung tissue; (E, F) t-SNE in lung tissue of RIPF rats

The abundance of each cluster of control versus RIPF cells is shown in Fig. 1, which demonstrates the relative abundance of each cell type genotype.

**Fig. 2:**
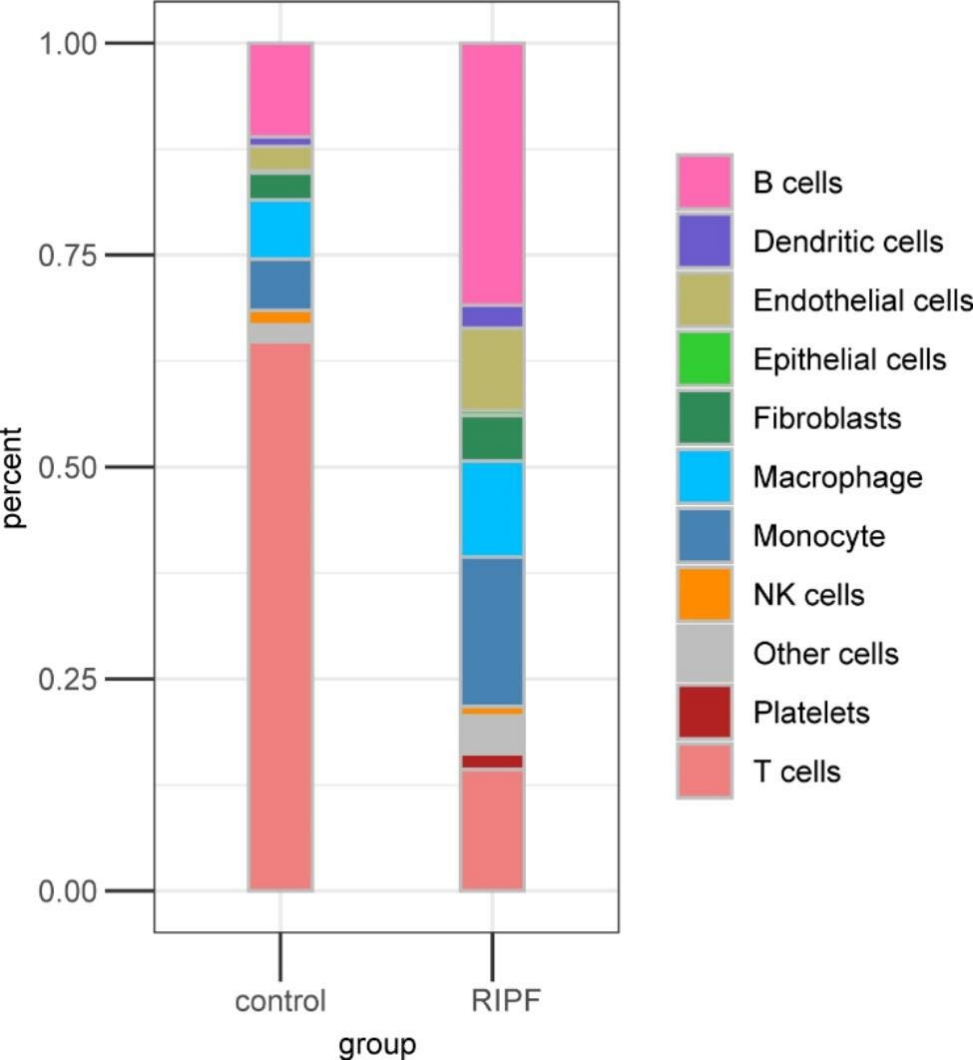
Relative abundance of each different cell type

In these scRNA-Seq data, the relative abundance of B and T cells in the RIPF group remarkably changed compared to that in the controls (Fig. 2); therefore, we initially analyzed these two cell types, and differentially expressed gene (DEG) analyses were performed on these data.

**Table 1:** Lung tissue cells are classified into 11 different cell types

Cell types	Markers
T cells	Cd3d, Cd3e, and Cd3g
NK cells	Klrd1, Gzma, Nkg7, and Il2rb
B cells	Ighd, Ms4a1, Cd79a, and Cd79b
macrophages	Adgre1, Cd68, and Lyz2
monocytes	S100a8 and S100a9
Dendritic cells	Cd83, Cd86, H2-Eb1, and H2-Ab1
platelets	Nrgn, Clec1b, Itga2b, and Itgb3
Epithelial cells	Scgb1a1, Sftpa1, Ager, and Krt7
Endothelial cells	Pecam1, Egfl7, Flt1, and Eng
fibroblasts	Col1a1, Col1a2, and Col3a1

### Analysis of DEGs of B cells

**Fig. 3:**
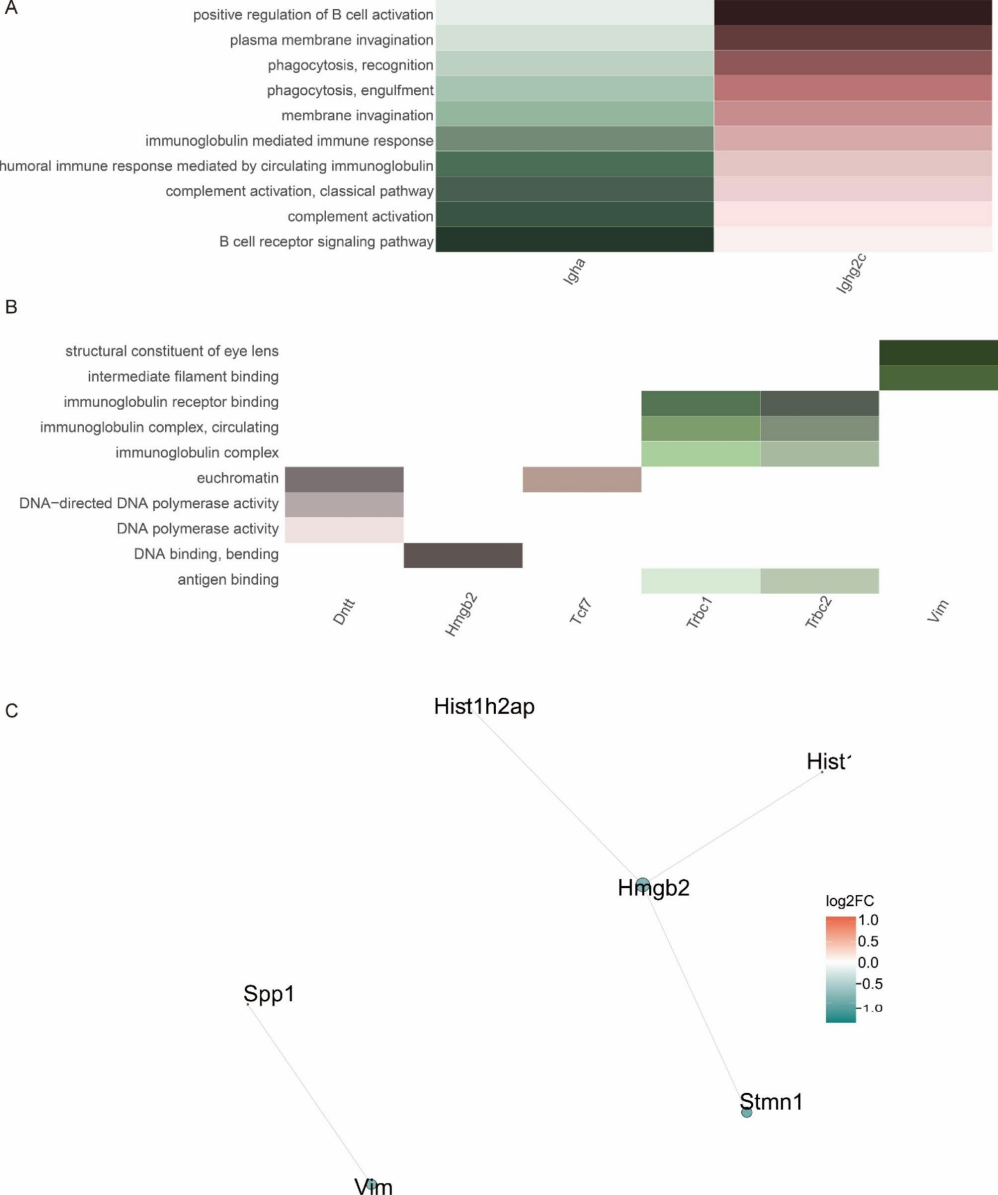
DEG analysis reveals pathway alterations in B cells between control and RIPF. representative GO (A, B) terms for DEGs in lung tissue cells; significance expressed as p-value calculated using BH exact test (P < 0.05) and expressed as -log_10_ (P value), visualizing the ten functions with the smallest p-values; (C) STRING analysis of DEG networks in B cells of RIPF. The shapes represent upregulated and downregulated information. Red represents upregulated gene networks, green represents downregulated gene networks, and the size of the dots represents the degree

Analysis of differential genes in B cells between the disease and control groups revealed significant differential expression of the following biological processes: structural constituent of the eye lens, intermediate filament binding, immunoglobulin receptor binding, immunoglobulin complex, circulating immunoglobulin complex, euchromatin, DNA-directed DNA polymerase activity, DNA polymerase activity, DNA binding, and antigen binding (Fig. 3B). These genes were enriched in the following pathways: positive regulation of B-cell activation, plasma membrane invagination, phagocytosis, recognition, phagocytosis, engulfment, membrane invagination, immunoglobulin-mediated immune response, humoral immune response mediated by circulating immunoglobulin, complement activation, classical pathway, complement activation, and B-cell receptor signaling pathway (Fig. 3A). The analysis revealed positive regulatory activation of B cells in the disease group, resulting in a significant increase in the number of B cells and a high correlation with immunoglobulins (Fig. 3C). Ionizing radiation can induce the secretion of immunoglobulins by activating B cells. Immunoglobulins reportedly enhance inflammation and promote the production of extracellular matrix and fibrogenic cytokines [22].

### Analysis of DEG of T cells

**Fig. 4:**
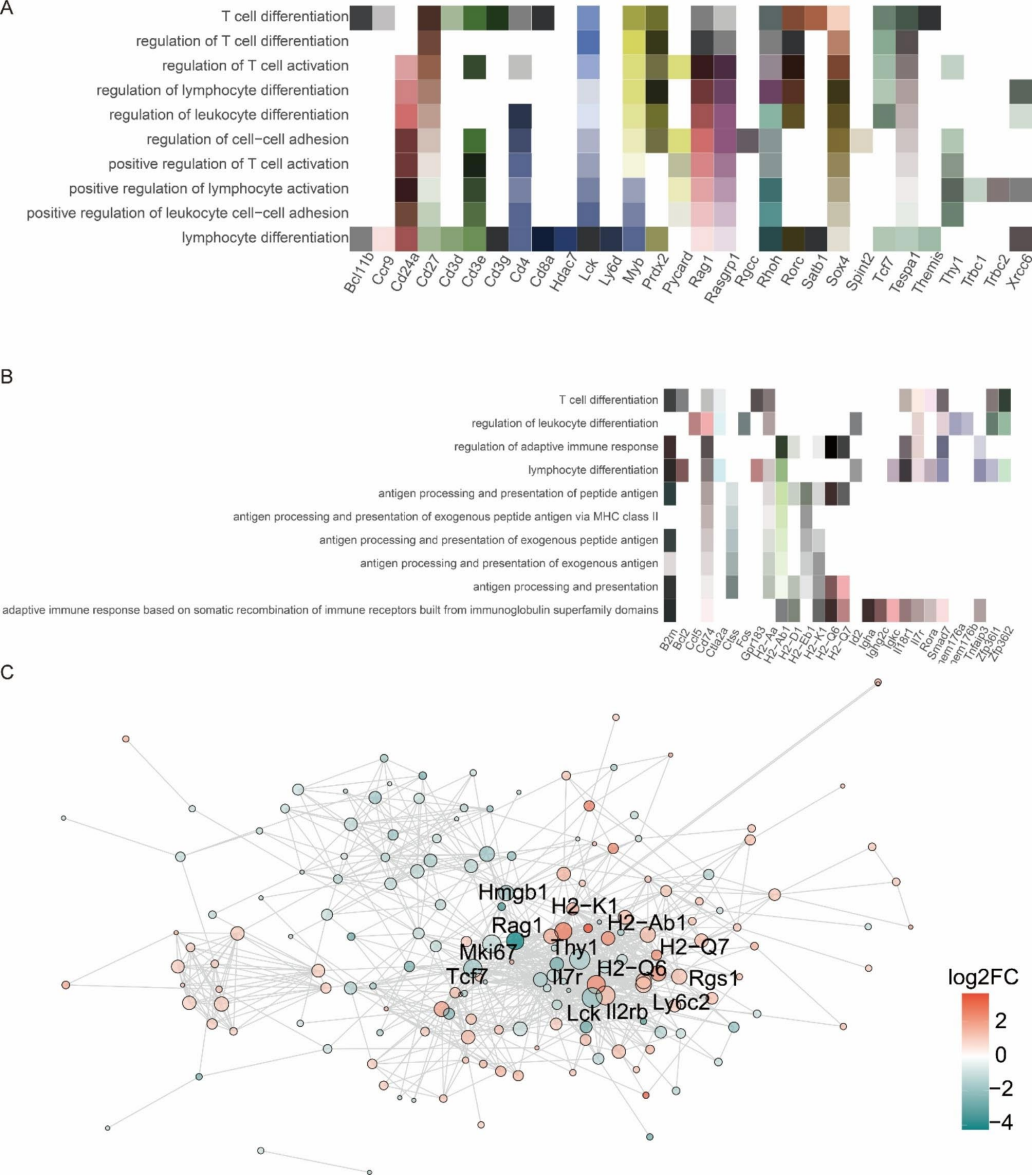
DEG analysis reveals pathway alterations in T cells between control and RIPF. representative GO (A, B) terms for DEGs in lung tissue cells; significance expressed as p-value calculated using BH exact test (P < 0.05) and expressed as -log_10_ (p value), visualizing the ten functions with the smallest p-values; (C) STRING analysis of DEG networks in B cells of RIPF. The shapes represent upregulated and downregulated information. Red represents upregulated gene networks, green represents downregulated gene networks, and the size of the dots represents the degree

The analysis of differential genes in T cells revealed that the following biological processes significantly differed: T cell differentiation, regulation of leukocyte differentiation, regulation of adaptive immune response, lymphocyte differentiation, antigen processing and presentation of peptide antigen, antigen processing and presentation of exogenous peptide antigen via MHC class II, antigen processing and presentation of exogenous peptide antigen, antigen processing and presentation of exogenous antigen, antigen processing and presentation, and adaptive immune response based on somatic recombination of immune receptors built from immunoglobulin superfamily domains (Fig. 4A). The top ten pathways with significant variation were as follows: T cell differentiation, regulation of T cell differentiation, regulation of T cell activation, regulation of lymphocyte differentiation, regulation of leukocyte differentiation, regulation of cell–cell adhesion, positive regulation of T cell activation, positive regulation of lymphocyte activation, positive regulation of leukocyte cell − cell adhesion, and lymphocyte differentiation (Fig. 4B). An apparent decrease in the number of T cells was observed in the lung tissue of rats following ionizing irradiation (Fig. 2). In summary, differentiation and activation signals in T cells and leukocytes and positive regulation of leukocyte cell–cell adhesion showed remarkable changes following ionizing radiation. Among the genes involved in these biological processes, we identified many cytokine-related genes, including CCL5, SMAD7, IL7r, IL18r1, TNFAIP3, and IL2rb, and immunoglobulin-related genes IGKC, IGHA, and IGHG2C (Fig. 4C).

### RIPF and cytokines

**Fig. 5:**
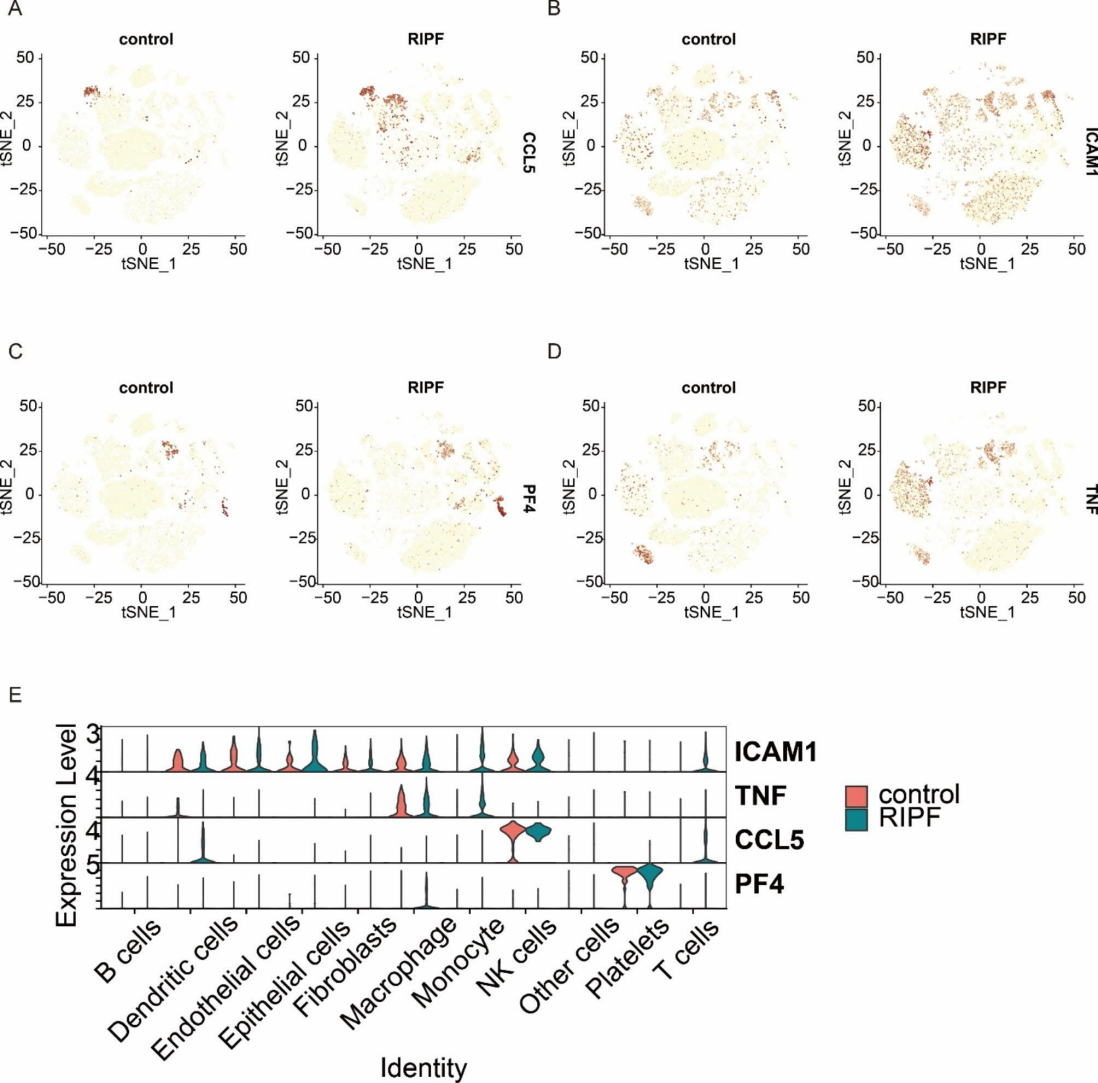
Cytokine expression levels in control and disease groups. (A–D) tSNE plots of differences in expression levels of four cytokines, CCL5, ICAM1, PF4, and TNF, between the normal and RIPF groups of cells. (E) Violin plots revealed the expression of four genes

The analysis of differences in gene expression between T cells and B cells indicated that a number of cytokines may be the underlying causative agent for the pathogenesis of RIPF. Consequently, RIPF single-cell transcriptome sequencing results were compared between those of disease and control groups, and the differential cytokines among them were selected for tSNE visualization. Four of these factors were visibly altered: CCL5, ICAM1, PF4, and TNFα (Fig. 5).

**Fig. 6:**
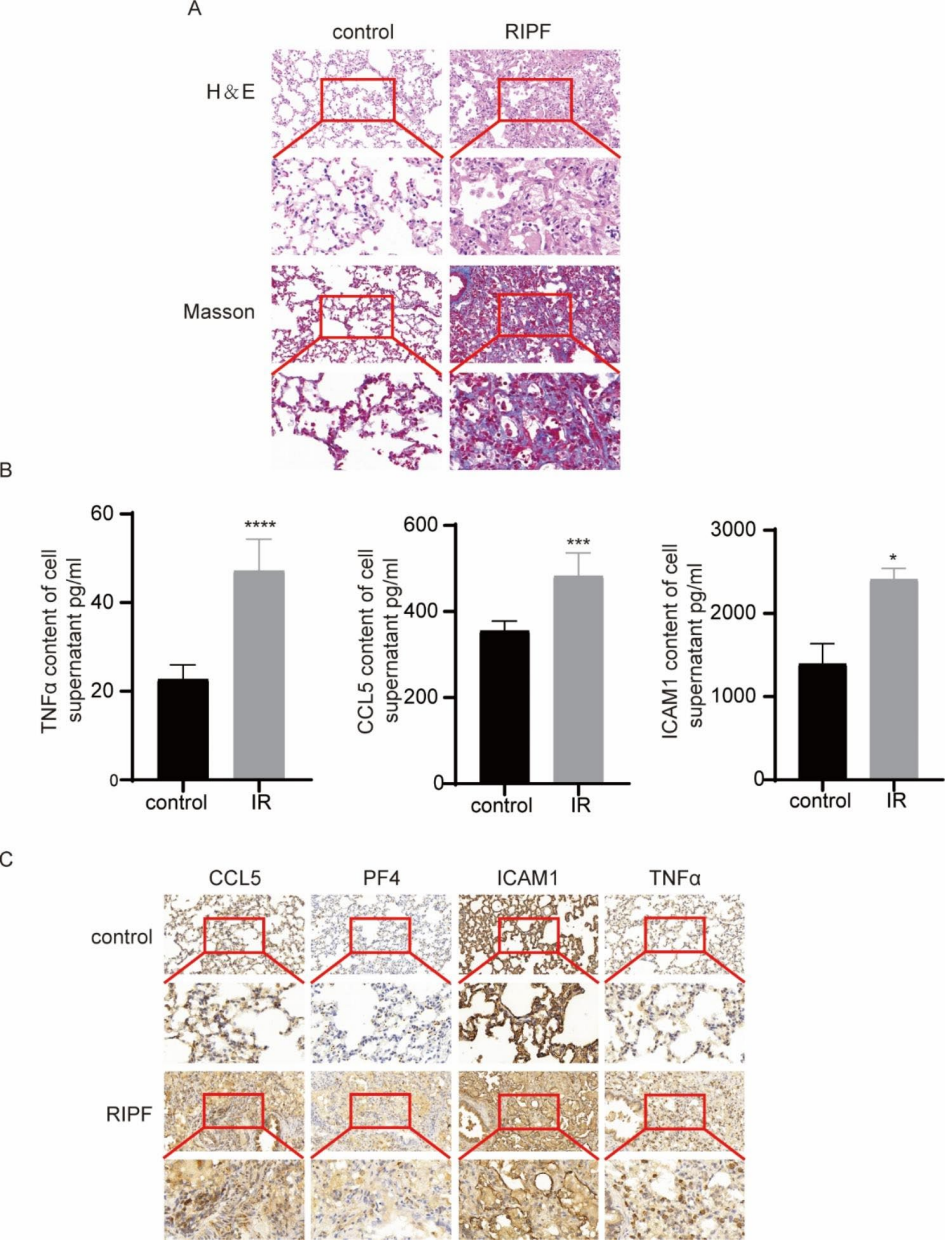
Changes in expression levels of the four cytokines in the irradiated and non-irradiated groups. (A) Assessment of areas of pulmonary fibrosis (H&E staining), intercellular and perivascular collagen deposition (Masson staining) in rats, n = 6; (B) ELISA showed upregulated cytokine expression in BESA-2B cells after ionizing radiation. (C) IHC staining of lung tissue showed higher levels of the four cytokines in the RIPF group than in controls. (*p < 0. 1, ***p < 0.001, ****p < 0.0001.)

Lung histopathological sections indicated that the alveolar structure of non-irradiated rats was normal. After 30 Gy irradiation, the alveolar septum was thickened and infiltrated by numerous inflammatory cells. Masson staining results were consistent with the pathological alteration seen in HE staining. The results demonstrated pronounced fibrous exudation of lung tissue in the irradiated group, exhibiting signs of RIPF (Fig. 6A).

ELISA results showed that the levels of CCL5, ICAM1, and TNFα in the supernatant of BEAS-2B cells evidently increased following ionizing radiation (Fig. 6B).

IHC staining showed that CCL5, ICAM1, PF4, and TNFα levels were significantly upregulated in the lung tissue of rats in the RIPF group (Fig. 6C).

## Discussion

RIPF is a serious complication, the mechanism of which is still unclear, thereby affecting the survival and prognosis of patients undergoing radiation therapy to shrink tumors. In biological processes, cytokines perform an overriding and significant role.

It has been previously demonstrated that chemokines are of considerable relevance in PF [23–26] and that C-C chemokines are involved in PF. CCL2 and CCL5 are upregulated in irradiated human lung epithelial cells, and inhibiting their expression constrains epithelial–mesenchymal transition and suppresses PF [27]. Previous studies have revealed that CCL2 facilitates fibrosis by supporting the monocyte/macrophage inflammatory response, fibroblast collagen synthesis, myofibroblast differentiation, and fibroblast recruitment and survival [28, 29], thereby protecting against PF in the absence of CCR2 signaling [30]. It has been suggested that CD4 + T lymphocytes of CCR2 are a subpopulation of CD4 + cells with anti-inflammatory and anti-fibrotic properties [31]. Our gene expression analysis revealed that CCL5 expression in T cells and dendritic cells was significantly upregulated in lung tissue after irradiation (Fig. 5A). ELISA results showed that CCL5 was upregulated in the cell culture supernatant of BEAS-2B cells after IR. IHC staining also demonstrated that CCL5 was upregulated in the lung tissue of RIPF rats (Fig. 6). Therefore, we hypothesized that CCL5(+) T cells may have an essential function in RIPF.

ICAM1 is a cell surface glycoprotein and a member of the immunoglobulin superfamily [32]. T cells and pro-inflammatory monocytes are recruited to the left ventricle via myocardial endothelial ICAM1, and ICAM1 upregulation may be associated with the stress overload induction of IL-1β and IL-6. When inflammatory infiltration occurs, leukocytes further release cytokines that promote cardiac inflammation, fibrosis, and cardiac insufficiency [33]. Recent studies have also reported that blocking ICAM1 can silence CD4IL4T cell differentiation in allergic rhinitis[34]. Therefore, it is reasonable to hypothesize that ICAM1 has a dearth of understudied connections with RIPF. We observed a highly detectable upregulation of ICAM1 in many types of cells in post-irradiation rat lung tissue, including monocytes, fibroblasts, endothelial cells, epithelial cells, and macrophages (Fig. 5B). ELISA results showed that ICAM1 level was upregulated in the cell culture supernatant of BEAS-2B cells after IR. IHC staining also demonstrated that ICAM1 was upregulated in the lung tissue of RIPF rats (Fig. 6). Therefore, we speculated that ICAM1 plays a little-known and critical role in RIPF; according to previous studies, this could be caused by T cell-induced inflammatory responses. However, this hypothesis requires further investigation.

Human platelet factor IV (PF4) is a heparin-binding protein contained in platelet alpha granules and is a member of the chemokine family, which is secreted upon platelet activation and is considered a marker of platelet activation[35, 36]. PF4 levels are elevated in the BALF of patients with scleroderma interstitial lung disease, and platelet activation occurs in the lungs [37]. In bleomycin-induced PF, platelet accumulation is associated with collagen deposition in the lungs and is involved in the development of interstitial lung disease [38]. This indicates that platelets play a crucial role in idiopathic pulmonary pathogenesis. We found significant upregulation of PF4 in platelets in post-IR rat lung tissue in tSNE, which may also suggest a major unexplained function of platelets in RIPF (Fig. 5C). IHC staining also demonstrated that PF4 is upregulated in the lung tissue of RIPF rats (Fig. 6), thereby supporting the hypothesis that PF4 is implicated in RIPF.

TNF is a pleiotropic inflammatory cytokine that plays a central role in many pathophysiological processes and can mediate tissue destruction by recruiting immune cells (e.g., neutrophils) [39]. The overexpression of TNF genes can lead to the development of pulmonary inflammation and fibrosis, and blocking TNF can dampen lung injury [40]. The depletion of CD4CD8 T lymphocytes prevented bleomycin-induced TNF levels and inhibited the extent of increased fibrosis. T lymphocytes led to impaired lung function and lung fibrosis by inducing an increase in lung TNF production [41]. Inflammatory mononuclear cells originate from the bone marrow and migrate to the lungs after radiation exposure [42]. The recruitment and activation of monocytes/macrophages are pivotal components of radiation-induced fibrosis [43]. We analyzed the changes in TNF levels in different cells and found that TNF levels decreased in macrophages and increased in monocytes. The principal biological function of TNF is TNFα; therefore, we assayed the level of TNFα in the cell supernatant after IR. ELISA results showed that the level of TNF-α was upregulated in the cell culture supernatant of BEAS-2B cells after IR. IHC staining results also demonstrated that TNF-α was upregulated in the lung tissue of RIPF rats (Fig. 6). Therefore, we assume that TNF plays a role in inducing RIPF in T cells and monocytes.

The process of radiation fibrosis is complex and the stimulated cells and the released cytokines differ depending on the time after irradiation. Radiation is the first to activate the immune system, which secretes cytokines leading to alveolar epithelial cell damage, vascular endothelial cell damage, and differentiation of fibroblasts into myofibroblasts[44]. Excessive activation of immune cells Th1 leads to acute radiation pneumonia and Th1 promotes cellular immunity by secreting Th1-related cytokines that mediate cytotoxic effects and local inflammatory immune responses, e.g., INF-γ, IL-2, IL-12[45–48]. Immune cells Th2 play a major role in the formation of late radiographic pulmonary fibrosis by inducing collagen synthesis to promote tissue remodeling and lung fibrosis, and Th2 stimulates B-cell proliferation and antibody production by secreting Th2-related cytokines, such as IL-4, IL-5, IL-6, IL-10, and IL-13, which play a major role in the formation of late-stage radiographic pulmonary fibrosis[49–52]. TGF-β, the key cytokine of fibrosis, is present at different levels at different stages after irradiation. After lung tissue was irradiated, the content of TGF-β in lung tissue increased significantly after 12 h, decreased to basal levels after 48 h, 72 h, and one week, and reached a maximum at 2 and 4 weeks, so the exposure of TGF-β expression has short and long-time duration increase after chest irradiation[53]. Therefore, exploring the levels of cytokines in lung tissue at different post-irradiation periods can help us to better understand the underlying mechanisms of lung fibrosis and thus to make regulatory interventions to alter the fibrotic process.

## Conclusions

In conclusion, through single-cell sequencing, we have gained further insights into the RIPF cell population and changes in the abundances of various cell types, with the most significant changes observed in the abundance of T- and B cells. We have also gained further insights into the role of cytokines in RIPF, based on the analysis of differential genes in these two cell types, confirming that four cytokines, CCL5, ICAM1, PF4, and TNF, play an important role in RIPF. Further investigation is required to assess their specific roles in RIPF.

## Data Availability

The datasets analyzed during the current study are available in the Sequence Read Archive (SRA, https://www.ncbi.nlm.nih.gov/sra) database, PRJNA768942.

## Abbreviations

ELISA: enzyme-linked immunosorbent assay
H&E: hematoxylin-eosin
IHC: immunohistochemical
PCA: principal component analysis
RIPF: Radiation-induced pulmonary fibrosis
RT: Radiotherapy
PF: pulmonary fibrosis
scRNA-seq: single-cell RNA-sequencing
